# Adaptations of interpersonal psychotherapy in psycho-oncology and its effects on distress, depression, and anxiety in patients with cancer: a systematic review

**DOI:** 10.3389/fpsyg.2024.1367807

**Published:** 2024-05-09

**Authors:** Ebba M. Laing, Jana M. Heinen, Rita Acebo de Arriba, Norbert Schäffeler, Stephan Zipfel, Andreas Stengel, Johanna Graf

**Affiliations:** ^1^Department of Psychosomatic Medicine and Psychotherapy, Psychooncology Division, University Hospital Tübingen, Tübingen, Germany; ^2^Comprehensive Cancer Center Tübingen-Stuttgart, University Hospital Tübingen, Tübingen, Germany; ^3^German Center for Mental Health (DZPG), Tübingen, Germany; ^4^Clinic für Psychosomatic Medicine and Psychotherapy, Klinikum Stuttgart, Stuttgart, Germany

**Keywords:** cancer, emotional adjustment, interpersonal counselling, interpersonal psychotherapy, mood disorders, oncology, psychosocial functioning, psychosocial intervention

## Abstract

**Objective:**

Patients with cancer experience significant psychosocial distress. Stressors include interpersonal difficulties like loneliness, isolation, thwarted belongingness, communication impediments, and conflicts. Interventions are required that address their specific psychosocial needs. Interpersonal Psychotherapy (IPT) is a promising concept for the treatment of psychosocial distress associated with cancer because it addresses patients’ interactions and role transformations. This review aims to provide an overview of the current evidence regarding interventions for patients with cancer based on IPT.

**Methods:**

A systematic review following PRISM guidelines was conducted, including randomized controlled trials of IPT-based interventions in patients with cancer, assessing effects on distress, depression, and anxiety.

**Results:**

Eight studies were included, sampling 390 patients in total. Seven out of eight studies assessed exclusively women with breast cancer. Two studies described IPT interventions and showed stronger improvement in depression and anxiety compared to TAU and equal improvement in depression compared to other psychotherapy interventions. Six studies described remote Interpersonal Counselling (IPC). One found remote IPC to be superior to control conditions regarding depression, and one found remote IPC to be superior to attention control, but not active control conditions. No study found remote IPC to be superior to control conditions regarding distress.

**Discussion:**

There are few randomized controlled trials of IPT for patients with cancer. Results regarding depression and anxiety are promising for in-person IPT, but mixed for remote IPC.

**Conclusion:**

The review suggests in-person IPT, but not remote IPC, may yield benefits for patients with cancer. Research on the subject is scarce, and to inform implementation of IPT interventions, research with diverse groups of patients with cancer is required.

**Systematic trial registration:**

PROSPERO, Identifier CRD42023410687.

## Background

Research of the past three decades has shown that patients with cancer experience psychosocial distress associated with the diagnosis, treatment, and adaptation to life post treatment of the disease. In a subset of up to 52% (Mehnert-Theuerkauf et al., 2018) of European patients with cancer, distress levels are clinically significant and warrant specific treatment. Symptoms of mood disorders are presented by 30 to 40% of patients with cancer (Holland et al., 2013).

A significant part of the distress experienced by patients with cancer derives from interpersonal difficulties. Patients must come to terms with fundamental changes in their social roles. Conflicts within their social networks may arise from contrasting adaptations to the new roles and divergent expectations of one another. Navigating the treatment context may elicit conflicts and require a whole new set of interpersonal skills.

High reported levels of distress in patients with cancer are associated with low treatment adherence and reduced quality of life, and may even increase mortality (Holland et al., 2013). High levels of perceived emotional and instrumental support, in turn, protect against the development of depressive symptoms in chronic physical illness and disability (Santini et al., 2015).

The high prevalence and detrimental effects of psychosocial distress call for interventions specifically tailored to the needs of patients with cancer. Due to their importance for patients with cancer, such interventions should aim at interpersonal factors of perceived distress.

Interpersonal Psychotherapy (IPT) is a modular concept of psychotherapy that focuses on the patients’ interactions and role transitions within their social context. Interpersonal Psychotherapy was first proposed by Klerman (1984). Its key element is understanding depression as an interpersonal phenomenon, closely intertwined with social roles and relationships. It uses the concept of the “sick role” (Parsons, 1951) to help the patient understand the social implications of depression. In detailed analyses of the patients’ current social network, the four “interpersonal problem areas” grief, interpersonal role disputes, role transitions and interpersonal deficits are explored. From these four areas, therapy goals are derived. In sessions, therapists utilize psychodynamic and cognitive-behavioral concepts and rely on established techniques such as reassurance, clarification of emotional states, improvement of interpersonal communication, and testing of perceptions and performance through interpersonal contact.

The fundamentals of IPT have been adapted in various forms to fit different cultural contexts, therapy settings, and patient groups [for an overview, see Ravitz and Watson, 2014]. IPT is one of the most thoroughly empirically validated forms of treatment of depression (Cuijpers et al., 2011). It has been shown to be the most effective out of six forms of psychotherapy for the treatment of depression in HIV seropositive patients (Cuijpers et al., 2016). It is also a well-established treatment for depression in elderly patients in primary care (Miller et al., 2001).

A counseling format of the program has been developed, addressing distress in patients who suffer from symptoms of depression and anxiety, but do not fulfill diagnostic criteria for a disorder (Interpersonal Counseling IPC; Weissman and Klerman, 1993). IPC is a shorter, more easily accessible format of IPT, and has been applied to various settings within primary health care. It has been shown that IPC reduces depressive symptoms in medically ill elderly patients (Mossey et al., 1996), in patients after myocardial infarctions (Oranta et al., 2012), and in women after pregnancy loss (Neugebauer et al., 2006).

At a first glance, there are several studies describing IPT for patients with cancer. However, there are great differences regarding important aspects of the studies’ designs and the interventions described. In order to help inform decisions about the implementation of an IPT program for patients with cancer, a thorough systematic overview of the literature is called for. The current review will examine the existing research and compile data concerning the following questions:

- How are the principles of Interpersonal Psychotherapy implemented in psycho-oncological settings?
- How does treatment based on Interpersonal Psychotherapy affect distress, depression, and anxiety in patients with cancer?

## Methods

### Registration

The review was conducted and reported in accordance with the Preferred Reporting Items for Systematic Reviews and Meta-Analyses (PRISMA). The review protocol was registered in advance with the International Prospective Register of Systematic Reviews (PROSPERO ID CRD42023410687).

### Eligibility criteria

Adhering to the PICOS scheme, eligibility criteria were defined as follows:

1. Population: adult patients currently or recently treated for cancer;
2. Intervention: Interventions based in theory and reasoning on IPT as described by Klerman (1984);
3. Comparators: comparisons to other forms of psychotherapy, counseling, or support; comparisons to no adjuvant therapy (treatment as usual); comparisons to pharmacological therapy for depression and/or anxiety;
4. Outcomes: Primary outcomes: Self-report and/or third-party assessments and/or clinical measures of depression, anxiety, and distress; additional outcomes: self-report of satisfaction and feasibility of the program, subjective improvements in mood and/or mental health.
5. Studies included: randomized controlled trials.

### Search strategy

Searches were conducted in the databases PubMed, Web of Science, PubPsych, and CINAHL on 04/06/23. The stem search term was “[Cancer OR Tumor OR Carcinoma] AND [Interpersonal Psychotherapy OR Interpersonal Support OR Interpersonal Coaching OR Interpersonal Counseling OR Interpersonal Therapy] AND [Distress OR Depression OR Anxiety OR comorbid OR psychiatric OR psychological OR symptom]”. Search terms were adapted to the respective search engines specific requirements. No restraints were used regarding publication date, publication type or language. An email alert was created in Web of Science to inform the reviewer of relevant articles published after the query. In addition, citations of identified records were searched for eligible studies.

### Selection process

Inclusion and exclusion criteria were developed using PICO criteria.

1. Population: studies targeting adult human subjects currently in treatment for cancer or with a recent cancer diagnosis (first and recurrent) were included. Studies targeting children or adolescents or adults with no cancer diagnosis were excluded.
2. Intervention: Studies using interventions based explicitly on IPT as described in the introduction were included. Adapted interventions such as IPC were included. Interventions lacking the foundation of IPT and/or not addressing the modules described by Klerman (1984) were excluded. Studies where no intervention was described were excluded.
3. Comparison: Only studies comparing the intervention to a different intervention or control condition were included.
4. Outcomes: Studies assessing the primary outcomes depression, anxiety, and distress before and after the intervention via self-report, third-party assessments and/or clinical measures were included, as were studies assessing the additional outcomes satisfaction with the intervention, and subjective improvements in mood and/or mental health and/or patient experience via self-report, third-party assessment and/or clinical measure.

Only articles containing data from one or more patients and descriptions of original studies were included. Reviews were to be assessed for additional sources, but were not considered for data retrieval.

**Figure 1:**
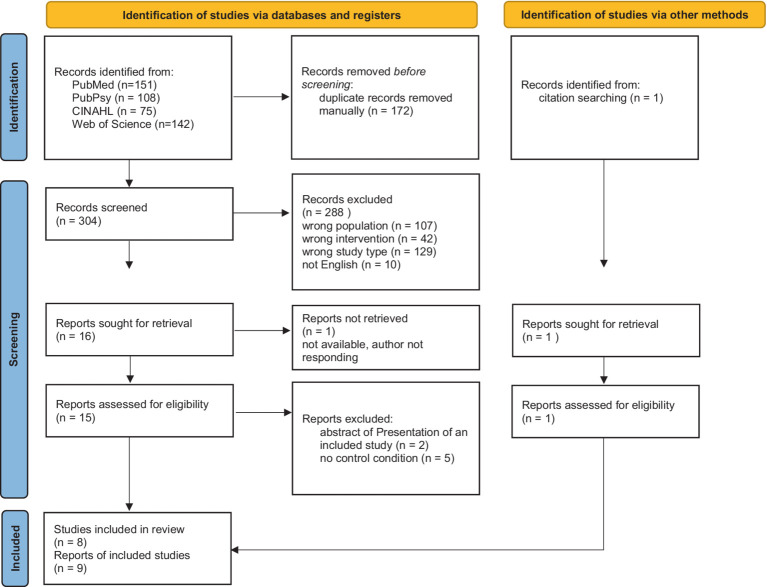
PRISMA 2020 flow diagram for new systematic reviews which included searches of databases, registers and other sources.

All articles retrieved in the database search were imported to Rayyan software (Ouzzani et al., 2016). Duplicates were detected by the software and reviewed and removed manually by one reviewer (EL). All remaining articles were screened considering title and abstract by two reviewers (EL and JH). Where the two reviewers disagreed, a third reviewer (JG) was consulted. The remaining articles were read in full by one reviewer (EL) and excluded if they did not match the criteria described above. The selection process is shown in full in the PRISMA flowchart (Figure 1).

### Extracted data

The following data were extracted from the studies included in the review: study type, treatment modality, targeted population, duration of intervention, adaptation of intervention, interventionists and their specific training measures, aims, focus, and modules of the intervention, number of participants, comparison condition, measures of stress, depression, and anxiety, key results of the pre- and post-intervention comparison regarding stress, depression, and anxiety. Missing data was reported as such.

### Assessment of risk of bias

The included studies were assessed for quality and potential bias using the revised Cochrane Risk of Bias tool (Sterne et al., 2019) for randomized trials. Assessment was conducted independently by two reviewers (EL, JH). Assessments were compared and in case of disagreement, a third reviewer (JG) was consulted.

## Results

### Study selection

Figure 1 visualizes the results of the screening process. 304 records were screened, and 16 were sought for retrieval. One record could not be obtained even after contacting the authors and the publishing journal (Dowse et al., 2000). One record was identified via citation screening of the records sought for retrieval (Badger et al., 2013b). Seven records were excluded after assessing for eligibility. Two records were describing the same study (Badger et al., 2005a,b). Because they provided complementary information on study procedures, both were included in the review. Thus, nine records of eight studies were included in the review.

### Sample characteristics

The included studies sampled a total of 390 patients with cancer. Of those, 36 were male and diagnosed with prostate cancer (Badger et al., 2011), the remaining 354 were female and diagnosed with breast cancer. Two studies included only patients with a cancer stage lower than four [Badger et al., 2005a, 2007). Among the other studies, cancer stages varied (stage IV = 9% (Blanco et al., 2019); *n* (stage IV) = 8 (Badger et al., 2011); *n* (stage IV) = 1 (Badger et al., 2013b); *n* (stage IV) ≈ 52 (Badger et al., 2020); *n* (stage III/IV = 26) (Badger et al., 2013a)]. One study did not report cancer stages for participants (Belay et al., 2022). Mean age in the male sample was 66.99 years (Badger et al., 2011). Mean age in the female samples ranged from 47.3 years (Badger et al., 2013a) to 58.5 years (Badger et al., 2013b). One author did not report mean ages but stated that the largest proportion of participants (42%) were aged 39 to 48 (Belay et al., 2022). All participants were treated in an outpatient setting.

### Assessment of risk of bias

Table 1 visualizes the results of the assessment of risk of bias. The assessment revealed high risk of bias for Badger et al. (2005a). A further record (Badger et al., 2005b), describing a subset of the sample, was consulted to supply additional information on the study procedures and concerns regarding randomization were ameliorated. Concerns regarding blinding of patients and providers of the intervention (D2) were disregarded due to the nature of the intervention. Thus, the study was included in the review.

**Table 1:** Results of analysis of risk of bias using the revised Cochrane risk of bias tool for randomized controlled trials ROB2.

Reference	Randomization Process	Deviations from Intended Intervention	Missing Outcome Data	Measurement of Outcome	Selection of Reported Results	Overall
Blanco et al. (2019)	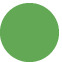	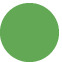	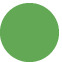	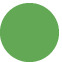	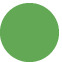	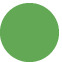
Belay et al. (2022)	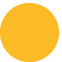	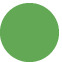	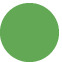	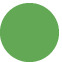	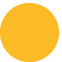	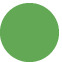
Badger et al. (2005a)	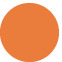	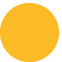	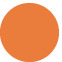	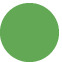	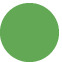	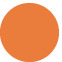
Badger et al. (2007)	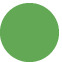	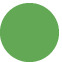	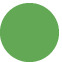	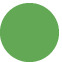	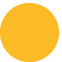	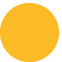
Badger et al. (2011)	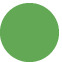	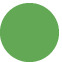	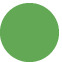	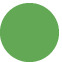	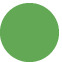	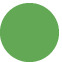
Badger et al. (2013a)	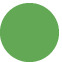	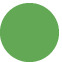	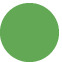	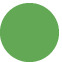	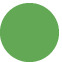	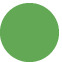
Badger et al. (2013b)	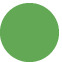	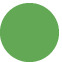	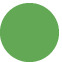	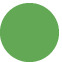	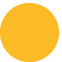	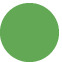
Badger et al. (2020)	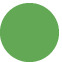	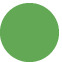	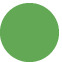	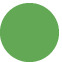	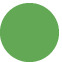	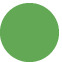

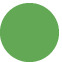
 Low Risk, 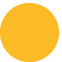 Some Concerns, 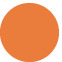 High Risk.

### Interventions

Two of the included studies were using IPT for their intervention (Blanco et al., 2019; Belay et al., 2022). The six remaining studies all used IPC, and all of them were conducted by the research group around principal investigator Badger et al. (2005a, 2007, 2011, 2013a,b, 2020).

Table 2 shows the characteristics of the interventions in detail. Table 3 shows descriptions of the composition of the interventions.

**Table 2:** Characteristics of interventions used in the studies reviewed.

Interpersonal Psychotherapy						
Reference	Modality	Target Population	Duration	Adaptation	Interventionists	Specific Training of Interventionists
Blanco et al. (2019)	In person, manualized	Breast cancer patients with current diagnosis of depression (SCID)	12 × 45 min	Bilingual (English, Spanish)	Psychologist, psychiatrist, social worker, treatment-specific psychotherapists	Treatment of two pilot cases, sessions audiotaped and supervised weekly by experts
Belay et al. (2022)	In person, manualized	Breast cancer patient with symptoms of anxiety and depression (HADS)	4–6 × 30–60 min	Ethiopian culture, health care context	Trained therapists	Not reported

Interpersonal Counselling						
Reference	Modality	Target Population	Duration	Adaptation	Interventionists	Specific Training of Interventionists
Badger et al. (2005a)	via telephone, manualized	Breast cancer patients and informal caregivers (separately)	6 sessions, avg. 32.9 min	Cancer, telephone	Master’s prepared clinical nurse specialists in psychiatric-mental health nursing with additional oncology training	32 h of didactic content (intervention protocol, theoretical aspects of the intervention, cancer treatment information, scientific literature), additional supervised counselling practice, sessions tape-recorded and reviewed, supervision
Badger et al. (2007)	via telephone, manualized	Breast cancer patients and informal caregivers (separately)	6 sessions, avg. 34 min	Cancer (informational support), telephone	Psychiatric nurse counsellor with oncology expertise	34 h of didactic content (intervention protocol, theoretical aspects of the intervention, cancer treatment information, scientific literature), additional supervised counselling practice, sessions tape-recorded and reviewed, supervision
Badger et al. (2011)	via telephone, manualized	Prostate cancer patients and informal caregivers (separately)	8 sessions, avg. 31 min	Cancer, telephone	Master’s prepared nurse or social worker with psychiatric and oncology expertise	Not reported
Badger et al. (2013a)	via telephone, manualized	Breast cancer patient and informal caregiver (separately)	8 sessions, avg. 29 min	Cancer, telephone, Latina bicultural context, bilingual	Trained social worker (bicultural, bilingual)	Sessions recorded and reviewed by experts to establish >90% adherence to protocol
Badger et al. (2013b)	via telephone or videocall, manualized	Breast cancer patients and informal caregivers (separately)	8 sessions, avg. 30 min	Cancer, telephone, bilingual (English, Spanish)	Social worker	Not reported
Badger et al. (2020)	via telephone, manualized	Breast cancer patients and informal caregivers (separately)	8 sessions, avg. 30 min	Cancer, telephone, Latina bicultural context, bilingual	Master’s prepared bilingual, bicultural social worker	24 h of training, including conducting and reviewing mock sessions, sessions audiotaped and reviewed to ensure >90% adherence to protocol

SCID, Structured Clinical Interview for DSM-IV; HADS, Hospital Anxiety and Depression Scale.

**Table 3:** Composition of the interventions.

Interpersonal Psychotherapy			
References	Objectives	Focus	Modules
Blanco et al. (2019)	Improvement in depressive symptoms	Crisis in role functioning or social environment IPT problem areas: loss, change, disagreement, interpersonal deficits	Not reported
Belay et al. (2022)	Improvement in depressive symptoms	IPT problem areas: loss, change, disagreement	Beginning phase: help patients to understand their problems, symptoms, explanatory model, and psychosocial supports; establishment of therapeutic relationship, provision of feedback, identification of IPT problem areas Middle phase: underlying interpersonal problems, helping patients to connect with support, extension of therapeutic focus to other areas linked to current distress Final phase: review of patients’ efforts and progress, contingency plans, processing old losses, processes that might be mistaken for original symptoms

Interpersonal Counselling			
References	Objectives	Focus	Modules
Badger et al. (2005a)	Decrease psychological distress	Current key relationship issues (grief, interpersonal role disputes, role transition and interpersonal deficits)	Interpersonal role disputes Social support Awareness and management of depressive symptoms Role transitions Cancer education
Badger et al. (2007)	Improve adjustment to cancer	Social support behaviours Interpersonal communication Current key relationship issues (grief, interpersonal role disputes, role transition and interpersonal deficits)	Interpersonal role disputes Social support Awareness and management of depressive symptoms Role transitions Cancer education
Badger et al. (2011)	Reduce psychological distress provide information about the illness and its effects	Social support behaviours Interpersonal communication	Identification of interpersonal problem areas Establishment of treatment contract Definition of symptoms within an interpersonal context Mood and affect management Emotional expression Interpersonal communication and relationships Social support Cancer education
Badger et al. (2013a)	Improvement of the quality of the relationship between survivors and other key players in the cancer experience normalization of patients’ experience and reactions	Social support behaviours Interpersonal communication	Mood and affect management Emotional expression Interpersonal communication and relationships Social support Cancer information
Badger et al. (2013b)	Improvement of quality of life	Social support behaviours Interpersonal communication	Mood and affect management Emotional expression Interpersonal communication and relationships Social support Resources and referral to support services
Badger et al. (2020)	Reducing psychological distress Improving quality of life normalizing the participants’ experience and reactions	Social support behaviours Interpersonal communication	Mood and affect management Emotional expression Interpersonal communication and relationships Social support Cancer information Resources and referral to support services

#### Execution

All interventions were reported to be manualized. They had all been adapted to the target group, either for cultural context or for the oncology setting and the remote delivery.

They were all delivered one-on-one. All studies except one (Belay et al., 2022) reported conducting specific training for providers of the intervention and thorough supervision of the sessions. While all IPT interventions were conducted in person, all IPC interventions were conducted remotely via phone, and in one study additionally via videocall^18^. IPT sessions lasted longer than average IPC sessions. IPT was provided mainly by specifically trained psychotherapists, while IPC was provided by nurses and social workers.

#### Objectives

IPT studies were targeting individuals with a cancer diagnosis and comorbid psychiatric symptoms (depression, anxiety), while in IPC studies psychiatric comorbidity was not a requirement for inclusion. Accordingly, IPT interventions aimed at improving the patients’ depressive symptoms. Aims of IPC interventions all centered around reducing psychological stress for patients with cancer but varied in more specific aspects.

#### Composition

IPT sessions focused on standard IPT problem areas (loss, change, disagreement, interpersonal deficits). One report referenced the IPT manual used and described its core modules, including psycho-education elements, establishment of a therapeutic relationship, attending to IPT problem areas, facilitating support, and final sessions including reflection of progress and relapse prevention measures (Badger et al., 2005a). One report gave no specific descriptions of the IPT manual used (Blanco et al., 2019). The IPT records did not clarify if or how specific cancer information was included in the sessions. IPC sessions focused mainly on the patients’ social support behaviors and interpersonal communication, with two studies also explicitly targeting the IPT problem areas. All IPC interventions included specific cancer information, except one (Badger et al., 2013b), which provided information about resources and referral to support resources instead. Descriptions of interventions modules and contents varied between IPC studies within the same research group. Upon request, T. Badger confirmed that the same core manual had been used in all studies conducted in the research group (personal communication, June, 2023). Modules included mood and affect management, interpersonal communication, interpersonal relationship, social support, and emotional expression, interpersonal role disputes, and awareness and management of depressive symptoms.

### Outcomes

Outcomes and key results are summarized in Table 4.

**Table 4:** Outcomes reported.

Interpersonal Psychotherapy							
Reference	Sample size n	Control Condition	Schedule	Outcomes	Measures	Key Results	
						Within-Group Comparison	Between-Groups Comparison
Blanco et al. (2019)	46	(a) Brief Supportive Psychotherapy (b) Problem Solving Therapy	T0: baseline T1: week 12	Stress	Not assessed	–	–
				Depression	17-Item Hamilton Depression Scale	Depression: effect size *d* = 1.07 response rate 35% (improvement of ≥50%) remission rate 25% (score ≤ 8)	No significant group × time interaction
				Anxiety	Not assessed	–	–
Belay et al. (2022)	57	Treatment as Usual	T0: baseline T1: week 8–10	Stress	Not assessed	–	–
				Depression	Hospital Anxiety and Depression Measurement Scale	Significant improvement (*p* < 0.001)	Significant group x time interaction with greater improvement in the intervention group than in the control group (*p* < 0.001)
				Anxiety		Significant improvement (*p* < 0.001)	

Interpersonal Counselling							
Reference	Sample Size *n*	Control Condition	Schedule	Outcomes	Measures	Key Results	
						Within-Group Comparison	Between-Groups Comparison
Badger et al. (2005a)	24	Attention control: weekly calls (8 min, no counselling)	T0: baseline T1: week 6 T2: week 10	Stress	25-Item Index of Clinical Stress	Significant improvement over time (*p* = 0.05)	No significant group × time interaction
				Depression	20-item Center for Epidemiological Studies—Depression Scale	No significant main effect for time (*p* = 0.19)	No significant group x time interaction
				Anxiety	Not assessed	–	–
Badger et al. (2007)	38	(a) self-managed exercise protocol and weekly phone calls (10 min); (b) attention Control: written cancer education and weekly phone calls (7 min, no counselling or exercise protocol)	T0: baseline T1: week 6 T2: week 10	Stress	Not assessed		
				Depression	20-item Center for Epidemiological Studies—Depression Scale	No significant main effect for time (*p* = 0.08)	No significant group x time interaction
				Anxiety	Composite score on 1–10 scale of 4 items of the PANAS, one item from SF-12 and 3 items from Index of Clinial Stress	Significant improvement over time (*p* < 0.001)	Significant group x time interaction (*p* = 0.01) with greater improvement in TIP-C and exercise group than in attention control group from T0 to T1, but not T1 to T2
Badger et al. (2011)	36	Telephone Health Education	T0: baseline T1: week 8 T2: week 16	Stress	10-item Perceived Stress Scale	No significant main effect for time	Significant group x time interaction with greater improvement in the control group than in the intervention group (*p* < 0.001)
				Depression	20-item Center for Epidemiological Studies—Depression Scale	No significant main effect for time	Significant group x time interaction with greater improvement in the control group than in the intervention group (*p* < 0.001)
				Anxiety	Not assessed	–	–
Badger et al. (2013a)	40	Telephone Health Education	T0: baseline T1: week 8 T2: week 16	Stress	10-item Perceived Stress Scale	Significant improvement over time (*p* < 0.001, *η*^2^ = 0.37)	No significant effect for group × time
				Depression	20-item Center for Epidemiological Studies - Depression Scale	Significant improvement over time (*p* < 0.001, effect size *η*^2^ = 0.3)	No significant effect for group × time
				Anxiety	20-item State–Trait Anxiety Inventory	Significant improvement over time (*p* < 0.01 *η*^2^ = 0.18)	No significant effect for group × time
Badger et al. (2013b)	33	Telephone Health Education	T0: baseline T1: week 8 T2: week 16	Stress	Not assessed	–	–
				Depression	20-item Center for Epidemiological Studies - Depression Scale	Significant decrease in symptoms for all groups (*p* < 0.001)	No significant group x time interaction
				Anxiety	Not assessed	–	–
Badger et al. (2020)	116	Telephone Health Education	T0: baseline T1: week 8 T2: week 16 T3: week 24	Stress	10-item Perceived Stress Scale	No significant main effect for time	No significant group x time interaction
				Depression	Patient Reported Outcome Measurement Information System	Significant improvement (*p* = 0.04) at T1, but not at T2 and T3	Significant group x time interaction at T1 (p = 0.04) with greater improvement in TIP-C group than in control group
				Anxiety		No significant main effect for time	No significant group x time interaction

#### IPT studies

Both IPT studies assessed depressive symptoms before treatment and after treatment or before and a month after treatment. There was significant within-group improvement of symptoms of depression reported in both studies (Blanco et al., 2019; Belay et al., 2022). One study assessed symptoms of anxiety and reported significant within-group improvement (Belay et al., 2022). Comparing IPT to Treatment as Usual, they found significant differences between the groups with participants in the IPT group scoring lower for depression and anxiety than participants in the TAU group (Belay et al., 2022). One study, comparing IPT to Problem Solving Therapy and Brief Supportive Psychotherapy, reported no significant group effect for symptoms of depression (Blanco et al., 2019).

#### IPC studies

The IPC studies reported mixed results.

##### Distress

Four out of six studies assessed distress. Two reported significant within group improvement (Badger et al., 2005a,a). Three reported no significant group difference comparing IPC to attention control and telephone-delivered health education (Badger et al., 2005a, 2013a, 2020). One study reported significant differences between groups with the telephone-delivered health education control group outperforming the IPC group (Badger et al., 2011).

##### Depression

Six out of six studies assessed depressive symptoms. Three of them reported significant within group changes (Badger et al., 2013a, 2013b, 2020). One reported significant between group differences with IPT outperforming the telephone-delivered health education control intervention post-intervention and at first follow-up, but not at second follow-up 4 months after the intervention (Badger et al., 2020). Four studies report no significant differences between groups, using attention control (Badger et al., 2005a, 2007), exercise protocols (Badger et al., 2007) and telephone-delivered health education (Badger et al., 2013a,b) for control interventions. One study reported significant differences between groups with the telephone-delivered health education control group outperforming the IPC group (Badger et al., 2020).

##### Anxiety

Three out of six studies assessed symptoms of anxiety. Two reported significant within group changes with one of them using a non-standardized composite measure of anxiety (Badger et al., 2007, 2013a). One reported significant between group differences with active treatments (exercise protocol, IPC) outperforming attention control post-treatment, but not at 1 month follow-up (Badger et al., 2007). Two reported no significant group difference comparing IPC to telephone-delivered health education (Badger et al., 2013a, 2020).

## Discussion

This review summarizes studies evaluating interventions based on IPT (Klerman, 1984) in oncological patients. The retrieved studies were reviewed for characteristics of the interventions and for effects of the treatments on the patients’ psychological distress, anxiety, and depression.

Nine records were included, sampling 390 patients in total, 354 of them women with breast cancer and 36 men with prostate cancer. Seven of the records described IPC interventions and two of the records described IPT interventions.

The IPT studies found that IPT leads to significant reduction in symptoms of depression and anxiety. Improvement in the IPT groups was greater than under treatment as usual, and equal to improvement under other forms of psychotherapy.

IPC was found to be superior to attention control conditions in one study for reducing symptoms of depression and in another study for reducing symptoms of anxiety. IPC was found to be equal to active control conditions in one study. Improvement under IPC was significant, but not superior to control conditions in two studies for reported distress, and in one study for symptoms of anxiety and depression. Improvement was not significant under IPC in two studies for distress, in three studies for symptoms of depression, and in one study for symptoms of anxiety. One IPC study found that IPC was less effective than remote health education in reducing distress and depression. This sample was the only one that consisted of men with prostate cancer.

The studies of IPT and IPC in oncological settings reaffirm that interventions based on IPT are versatile in their realization and can be adapted to fit patients’ specific logistical needs, schedules, and surroundings.

In two studies, interventions were compared to active treatments: IPC to an exercise protocol (Badger et al., 2007), and IPT to Brief Supportive Psychotherapy and Problem Solving Therapy (Blanco et al., 2019). In both cases, all active conditions yielded similar results. This is coherent with theories that non-specific factors of psychotherapy contribute more to its effect than specific content factors (Grawe et al., 1994). It could also be argued that regarding such non-specific factors, telephone IPC was too similar to telephone health education to yield additional benefits and was therefore not always superior to that control condition.

The effectiveness of IPT in reducing anxiety and depression in patients with cancer is consistent with its strong effects in the treatment of HIV-seropositive patients (Cuijpers et al., 2016). The core themes of IPT are very important in developing and maintaining depressive symptoms for both patient groups. Both may be reminded of past losses when confronted with the possibly fatal diagnosis of cancer or a HIV infection. Both must manage role changes, giving up roles that they can no longer fill due to physical or cognitive impairments, and possibly taking up new roles as patients in need of care. Both HIV-seropositive patients and patients with cancer depend heavily on their interpersonal skills. In reviewing the patients’ handling of disagreements and interpersonal role disputes, IPT enables them to connect with their social network while communicating personal boundaries and needs. In focusing on interpersonal deficits, patients can improve their ways of engaging with the medical system and activate social support by learning how to talk about the diagnosis, its implications, stigma, and their needs and fears.

The results regarding in-person IPT in patients with cancer are also coherent with the effectiveness of IPT in treating depression in medically ill elderly people (Mossey et al., 1996). The risk of getting cancer increases with age, so there is significant overlap between the two groups of elderly people with depression and patients with cancer with depression. Losses within the social network may be a very important factor contributing to depressive symptoms, especially for elderly patients with cancer. There are also many similarities in the role changes that elderly people and patients with cancer face, since these changes arise from declining physical or cognitive abilities. And just like for physically ill patients, disagreements and interpersonal deficits are an impediment for many elderly people in accessing the social support they require.

The primary purpose of the IPC interventions is to reduce distress in patients with cancer. Yet, the results regarding distress are not conclusive. Even in the study with the largest sample size (*n* = 116), no significant improvement over time could be detected (Badger et al., 2020). There are a few possible explanations for the lack of clear overall evidence supporting IPC. For one, IPC sessions were usually shorter and fewer than the sessions in IPT trials. However, duration and frequency of sessions were in accord with IPC guidelines and most other trials investigating IPC reported by Weissman et al. (2014), and were expected to suffice to reach the aims of IPC.

It is possible that the mixed effects of IPC interventions are due to ceiling effects of distress reduction in patients with cancer. With a possibly life-threatening diagnosis of breast cancer, a certain level of distress, depression, and anxiety may persist past a 6 month-follow-up in all patients regardless of interventions. Accordingly, mean scores for depression and anxiety post-intervention in the IPT trials were still within the range of “subthreshold” symptoms. Symptom burden persisted, although at a significantly lower level. Since in the IPC trials, pre-intervention scores of distress, depression and anxiety were rather low in most samples, expecting further improvement towards total remission may be unrealistic.

It is possible that samples with high baseline scores improved more strongly under IPC than samples with low baseline scores. For symptoms of depression, this is illustrated by the fact that out of the five studies that used the 20-item Centre for Epidemiological Studies – Depression Scale, only two samples scored clearly above the recommended cutoff for “mild depression” (score ≥ 16 out of 60) at baseline (Badger et al., 2013a,b). In those samples, symptom improvement was significant over time. One sample scored only slightly above the cutoff (at 16.44) (Badger et al., 2007), and two samples scored below the cutoff (Badger et al., 2005a, 2011), and in those sample changes were not significant. A similar tendency can be observed for baseline scores of distress in three studies (Badger et al., 2011, 2013a, 2020), but the baseline differences between samples are marginal here. Measures of anxiety were not compatible across studies.

In addition, one of the IPC samples was the only male sample to be reviewed (Badger et al., 2011). Here, the remote health education intervention led to greater improvement in distress, depression and anxiety than the remote IPC. The positive effects of in-person IPC have been shown in previous studies on male and female patients with physical illnesses other than cancer (Weissman et al., 2014), and the results could just be attributed to the small sample size in this study (*n* = 36). However, the lack of improvement might imply that male patients struggled to open up about their mental health and interpersonal issues in this remote IPC setting.

The efficacy of in-person IPC interventions in reducing symptoms of depression and anxiety has been shown in physical illness other than cancer, and the discrepancy with results of the telephone-delivered IPC trials in patients with cancer indicates that remote IPC might be less effective, at least in this population. IPC relies heavily on the interventionist evaluating and reflecting a patient’s individual symptoms and their idiosyncrasies in verbal and nonverbal communication and interaction (Weissman et al., 2014). Telephone IPC, with no visual contact between interventionist and patient, may not be able to provide such feedback. In addition, like all other psychological interventions, therapeutic relationship is an important factor in IPC. A strong therapeutic relationship will help the patient speak openly about intimate issues and trust the advice and propositions of the interventionist. It will also allow the interventionist to challenge the patient to explore change without breaking their trust (Schramm, 2017). From the results of the reviewed studies, it could be inferred that the relationship with the interventionist cannot be established as quickly or as deeply via the telephone as in-person. It is to be investigated whether IPC interventions via videocall, in providing visual contact between provider and receiver of the intervention, increases the interventions effect on distress, depression, or anxiety. One of the IPC studies compared IPC via telephone to IPC via videocall and found no significant difference in effect between the two groups^18^. Both samples, however, were very small and observations cannot be generalized.

### Study limitations

Overall, research into the interventions’ effects on distress, anxiety, and depression in patients with cancer is scarce and limited to studies with small samples. They can only serve for preliminary and very cautious interpretation.

All studies of IPC were conducted under supervision of the same principal investigator at the same study center. Thus, bias regarding the studied population in cultural background and treatment variables, and regarding the conceptualization and implementation of the intervention is highly probable. This decidedly weakens generalizability of the findings regarding IPC.

Because there are no studies comparing (telephone) IPC to treatment as usual, it cannot be clearly determined if remote IPC is superior to natural improvement of distress, or symptoms of depression and anxiety in patients with cancer over time.

All IPC trials included separate remote IPC sessions for one primary caretaker of the patients with cancer. It could be supposed that the effect of interpersonal interventions is more pronounced when the intervention is offered to both parties. Therefore, results for the patients should be regarded with caution as they might be inflated due to the caretakers’ intervention.

Follow-up periods were rather short (max. 24 weeks) and therefore, effects beyond that scope may have been omitted. IPC may produce a prophylactic effect by preventing the onset of stronger symptoms of depression or anxiety. It also serves as triage to identify individuals with need for more or different interventions. Longer follow-up periods could help quantify those prophylactic effects and help evaluate the cost-effectiveness of IPC.

When investigating mental health of patients with cancer, the amount of time that has passed since the patient has received their diagnosis or treatment is also an influential factor. Natural adjustment to the new circumstances is to be expected to a certain extent, and may account for improvement during the trial. Unfortunately, time since diagnosis was not consistently reported across the reviewed studies and can therefore not be taken into account when comparing the results.

The review is further limited by two very important overall sample characteristics, gender and cancer site. Out of eight studies in this review, seven included exclusively female patients with breast cancer. Social roles and expectations are closely intertwined with gender, and gender roles should be taken into account when assessing and discussing interpersonal distress. Social and role functioning are affected in different ways in different cancer sites (Licht et al., 2021). Each cancer site requires a different treatment regimen, and some patients, e.g., with leukemia, may need long hospital stays and phases of isolation, while others, e.g., patients with lung cancer may receive their treatment in an outpatient setting. They incur different short- and long-term physical changes and disabilities. Some patients, e.g., with breast cancer, may be able to return to work after completing the treatment, while others, e.g., with brain tumors, mas be hindered from working due to physical or neurological impairments. The affected organ itself may play a role in social functioning. Breast- and ovarian cancer, for example, often incur struggles regarding the patients’ sexuality and self-perception. Thus, results assessed only in patients with breast cancer cannot be generalized to other patient populations.

### Clinical implications

At the current state of research, telephone-delivered IPC appears to yield no reliable benefit for patients with cancer. Studies of the intervention are few and generalization is impeded by severe limitations.

From the two studies eligible for this review, it appears that in-person IPT can be applied to and yield benefits for patients with cancer. To date, it is studied only in manualized one-on-one outpatient settings. Cancer-specific education materials are added, but the standardized IPT structure is maintained.

To inform health care providers whether implementing an IPT intervention will benefit their patients and justify the effort of training and supervision of interventionists, more research on the subject is called for. The short- and long-term effects of IPT and IPC interventions should be assessed where possible and compared to control conditions. In practice, clinicians are likely to encounter patients from different areas of oncology and of all genders. Thus, research is urgently required including patients of all genders and diagnosed with different cancer sites to further our understanding of the effect and applicability of IPT or IPC in psycho-oncology.

## Conclusion

At this stage, evidence supporting the benefits of IPC via telephone or videophone over control conditions cannot be considered sufficient to recommend application in the psycho-oncology context. Results of intrasubject improvement regarding distress, depression and anxiety are inconclusive and generalizability is limited.

Conceptually, IPT’s core elements of interpersonal conflict, interpersonal deficits, grief, and role transformations make it uniquely suited to alleviate distress, depression, and anxiety specifically in patients with cancer. By addressing depression and anxiety both as results of and as causes of difficulties in relationships, it allows patients to see themselves in constant interaction with their social network and provides them with the agency and the tools to break from their perceived isolation. By acknowledging grief and loss, it gives patients with cancer an opportunity to speak about fears and sorrows they may hesitate to share openly otherwise. Moreover, in discussing and aiding successful role transformations, it provides patients with cancer with a framework for the changes happening in their lives. As this review shows, randomized controlled trials of IPT in women with breast cancer support the feasibility and effectiveness of in-person IPT provided by trained therapists in reducing depression and anxiety in this population. As they are very few, effects should be investigated further, assessing larger and more diverse populations.

## Data availability statement

The original contributions presented in the study are included in the article/supplementary material, further inquiries can be directed to the corresponding author.

## Author contributions

EL: Writing – original draft, Writing – review & editing. JH: Writing – original draft, Writing – review & editing. RA: Writing – review & editing. NS: Writing – review & editing. SZ: Writing – review & editing. AS: Writing – review & editing. JG: Writing – review & editing.
